# Use of dietary diversity score as a proxy indicator of nutrient adequacy of rural elderly people in Sri Lanka

**DOI:** 10.1186/1756-0500-5-469

**Published:** 2012-08-29

**Authors:** Kumari Malkanthi Rathnayake, PAE Madushani, KDRR Silva

**Affiliations:** 1 Department of Applied Nutrition, Faculty of Livestock, Fisheries & Nutrition, Wayamba University of Sri Lanka, Makandura, 60170, Sri Lanka

**Keywords:** Validation, Dietary diversity indicators, Nutrient adequacy, Elderly

## Abstract

**Background:**

Macro and micro nutrient deficiencies are public health concerns in most developing countries including Sri Lanka, partly due to monotonous, cereal-based diet that lacks diversity. The objective of the study was to assess validity of food variety score (FVS), dietary diversity score (DDS) and dietary serving score (DSS) as indicators of nutrient adequacy of rural elderly people in Sri Lanka.

**Findings:**

A sample of 200 apparently healthy elderly people >60y of age were studied. A single 24 h recall was performed to compute dietary diversity indicators. Pearson’s correlation was used to assess the utility of FVS, DDS and DSS as indicators of nutrient adequacy. Sensitivity (Se) and specificity (Spe) analysis were done to determine the most appropriate cut-off points for using FVS and DDS to categorize elderly people with adequate nutrient intake. The average (standard deviation) of the food variety score, dietary diversity score and dietary serving score was 8.4 (2), 4.4 (0.9) and 11.4 (2.5), respectively. Mean adequacy ratio (MAR) of 12 nutrients was 0.39 (39%). Pearson’s correlation coefficients between MAR and FVS was 0.45 (*P* < 0.01), for DDS it was 0.48 ( *P* < 0.01) and for DSS it was 0.58 ( *P* < 0.01). When maximizing sensitivity and specificity, the best cut-off point for achieving 50% of MAR was about 9 and 4.5 for FVS and DDS, respectively.

**Conclusion:**

In conclusion, FVS, DDS and DSS were useful proxy indicators of nutrient adequacy of rural elderly people in Sri Lanka. Indeed, the performance of the indicators is improved when considering the quantities of food consumed.

## Findings

### Introduction

Dietary diversity (DD) has been universally identified as a key element of high quality diets. As dietary factors are associated with increased risk of chronic diseases and undernutrition, local and international dietary guidelines recommend to improve the diversity of the diet. Macro and micro nutrient deficiencies are public health concerns in most developing counties including Sri Lanka, due to monotonous, cereal-based diet that lacks diversity [1]. Furthermore, diverse diet reflects the nutrient adequacy of the diet [2]. Several studies have also shown that the overall nutritional quality of the diet is improved with diverse diet [3-5]. Therefore, diversity in the diet is important to meet the requirements for energy and other essential nutrients especially for those who are in the risk of nutrition deficiencies [6].

Elderly people are at a greater risk of nutritional deficiencies due to physiological changes associated with aging, acute and chronic diseases, financial and social status and functional decline [7]. Indeed, with the aging process, inevitable changes are occurred in each body organs, such as shifting body composition, changes in immune system and sensory changes [8]. Diverse diets have been shown to protect against chronic diseases such as cancers [9], as well as being associated with prolonged longevity [10] and improved health status [11].

Sri Lanka is a low-middle income country undergoing a rapid epidemiological and nutrition transition. With the improvement of healthcare facilities in Sri Lanka, the elderly population is increasing gradually over the past years. Therefore, the approach of evaluating total diet quality is important. Such methods should be valid as well as simple and inexpensive to practice in a developing country. Although, the researchers have indicated that the dietary diversity (DD) is a useful indicator of nutrient adequacy among children and adults [1], evidence is limited to support the utility in elderly populations. Nevertheless, quantifying and operationalizing DD is a challenging research task. Therefore, the aim of the present study was to assess validity of the dietary diversity score (DDS), a simple count of food groups consumed, food variety score (FVS), a simple count of food items consumed and dietary serving score (DSS), number of servings of different food groups in conformity with dietary guidelines as indicators of nutrient adequacy in rural elderly population in Sri Lanka. Moreover, we quantified the appropriate DDS and FVS cut-off points to use as indicators of inadequate nutrient intake. This study will address the need for a set of comparable validation studies for creating dietary diversity indicators to predict nutrient adequacy. Hence, the findings of this study could be used as rapid assessment tools for measuring diversity of the diet as well as indicators to monitor individual nutrition security.

## Methods

### Study area and subjects

This study was conducted as a cross sectional study during the period of March to July 2009. A sample of two hundred apparently healthy elderly people of > 60 years of age were selected randomly from the electoral list from Dankotuwa and Pannala divisional secretariats (DS) based on the elderly population living in two DS divisions of Puttlam and Kurunegala districts in Sri Lanka. Subjects with a prior history of cardiovascular diseases, diabetes and stroke were excluded because of possible changes in the diet.

### Dietary assessment

The food intake data of the subjects were collected by individual single 24-h food recall by a trained person in a random day to minimize day to day differences. A standard protocol was used to take 24 hour recall. The interviews included a detailed description of the foods eaten, the cooking method, and brand names (e.g. for milk consumed or other processed snack foods). The amount consumed by the subject was estimated by the respondent, expressed in terms of cups, table spoons, coconut spoons, size of box of matches, and other common household measures. The respondents were shown visual aids (photographs of servings) to assist them for accurate reporting of food intake. Nutrient intakes were computed using electronic database comprising nutrient compositions of Sri Lankan foods.

### Assessment of dietary diversity

Dietary diversity (DD) is defined as the number of different foods or food groups consumed in the previous day and it was measured using four methods, namely; Food variety score (FVS), Dietary diversity score (DDS), DDS-half serving and Dietary serving score (DSS).

### Food variety score (FVS)

This was measured using simple count of individual food items consumed over last 24 hour. When counting food items, we ignored condiments and beverages. Among the beverages we excluded only tea and coffee infusions.

### Dietary diversity score (DDS)

DDS was calculated by summing the number of unique food groups consumed during last 24 hour as described by Krebs-Smith et al. 1987 [12]. Food groups considered were cereals/roots, vegetables, fruits, legumes/lentils, meat/fish/egg & milk/dairy products. If an individual eat any quantity of any food group at least once per day, was taken into count. Therefore, DDS was calculated without considering a minimum intake for the food group.

### DDS-half serving

This was calculated applying a minimum consumption of half serving of respective food groups as described above.

### Dietary serving score (DSS)

Same six major food groups were considered for scoring system and a maximum score of 20 was allocated for these food groups as described below [10]. Vegetables, fruits and dairy groups were received maximum of 4 points for each two recommended servings and 4 points were received for 4 recommended servings of cereals/roots. Maximum of 2 points were received for each 1 recommended serving of plant and animal source of protein groups. Recommended servings were calculated by referring the Food Based Dietary Guidelines for Sri Lankans [13]. Table 1 shows the recommended servings for each food group and assigned scores.

**Table 1:** Recommended servings of each food group and assigned scores

**Food group**	**No of servings**	**Assigned scores**
Cereals/roots	4	4
Vegetables	2	4
Fruits	2	4
Legumes/lentils	1	2
Meat/fish/egg	1	2
Milk/dairy products	2	4
**Total**		**20**

### Assessment of nutrient adequacy

Nutrient adequacy was measured by computing Mean Adequacy Ratio (MAR). To compute MAR first Nutrient Adequacy Ratio (NAR) was calculated for the energy and 11 nutrients as given below.

### NAR

The NAR for a given nutrient is the ratio of a subject’s intake to the current recommended allowance for each sex and age category [3,14]. To estimate the nutrient adequacy of the diet, NAR was calculated for the energy intake and 11 nutrients.

(1) $$\text{NAR}=\frac{\text{Actual nutrient intake of a nutrient}\;\left(\text{per day}\right)}{\text{Recommended daily allowance of the nutrient}}$$

MAR

As an overall measure of the nutrient adequacy, the MAR was calculated as used by Hatloy et al. 1998 [3].

(2) $$\text{MAR =}\frac{\sum NAR\left(each\;truncated\;at\;1\right)}{\text{Number of nutrients}}$$

NAR was truncated at 1, so that a nutrient with a high NAR could not compensate for a nutrient with a low NAR.

Ethical clearance for this study was obtained from the Ethical Review Committee of the Wayamba University of Sri Lanka, and informed consent was obtained from the subjects before the data was collected.

## Results

### Characteristics of the sample

The mean age of the sample was 68 ± 7 years and the majority was females (70 percent). The majority of the elders had the education level of up to grade 8 only. More than 50 percent of elders depended on their family helps, especially from their children and their monthly income was lower than LKR 3000 per month.

### Dietary diversity indicators

#### Food variety score (FVS)

The mean FVS of the study sample was 8.4 while the theoretical maximum was 15.

#### Dietary diversity score (DDS) and DDS-half serving

**Figure 1:**
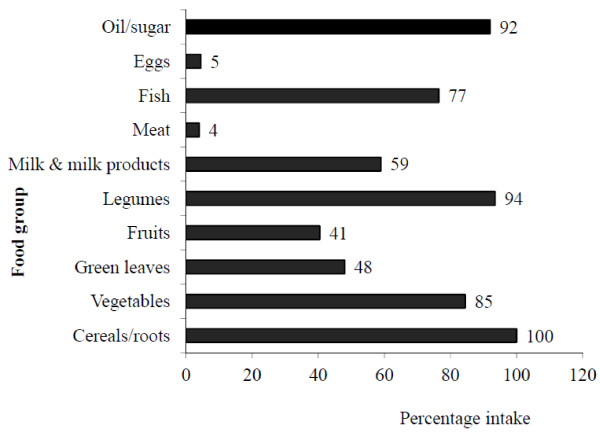
Consumption of different food groups by the elders during the previous day of the survey.

The mean DDS was 4.4 and decreased to 3.8, when a DDS-half serving size was applied and the theoretical range of DDS was from 1 to 6. Figure 1 shows the consumption of food from different food groups among the study population in the previous day of the survey. When considering the dietary diversity and consumption pattern of the sample, all the elders had used some kind of a cereal. Furthermore, a higher proportion had used oil/sugar and legumes and it was more than 90 percent. In addition, vegetables were also consumed by more than 80 percent of the sample. Nearly 60 percent of the elders had consumed at least one milk product during the previous day of the survey. Less than 50 percent of the study sample had consumed fruits and green leaves. Overall animal food consumption was not at the satisfactory level (Figure 1). Fish consumption was greater (77 percent) compared with other animal food sources i.e. meat and eggs in the sample.

#### Dietary serving score (DSS)

The mean DSS was 11.4 whereas the theoretical maximum was 20 for the DSS.

#### Nutrient adequacy

Mean adequacy ratio (MAR) of 12 nutrients of the study population was 0.39 (39 percent), whereas the diet that covers the recommended intake for all considered nutrients should have a 1.00. Table 2 shows the mean values for all dietary diversity indicators and nutrient adequacy.

**Table 2:** Dietary diversity and nutrition adequacy of the study population

**Dietary diversity indicator**	**Mean**	**SD**
FVS	8.4	2.0
DDS	4.4	0.9
DDS-half	3.8	1.0
DSS	11.4	2.5
MAR	0.39	0.1

#### Validation of dietary diversity indicators against nutrient adequacy

Table 3 shows the correlations between the different dietary diversity indicators and the nutrient adequacy expressed as NAR for each nutrient and MAR, as an overall score for the nutritional adequacy. All four dietary diversity scores correlated significantly with almost all nutrients except for vitamin D. Meanwhile, MAR, overall nutrient adequacy score, correlated strongly with all four dietary diversity scores. The NARs for many nutrients were more positively correlated with both FVS and DSS than DDS and DDS-half serving.

**Table 3:** Mean nutrient intakes (SD), NAR, correlations of NAR and MAR with FVS, DDS, DDS-half serving and DSS of the study population

**Nutrient**	**Mean intake**	**SD**	**NAR**	**Correlations**			
				**FVS**	**DDS**	**DDS -half**	**DSS**
Energy (kcal/d)	951	300	0.49	0.35^*^	0.25^*^	0.26^*^	0.32^*^
Protein (g/day)	23.4	8.9	0.50	0.35^*^	0.29^*^	0.26^*^	0.30^*^
Calcium (mg/d)	218.3	118.9	0.17	0.33^*^	0.28^*^	0.29^*^	0.39^*^
Iron (mg/d)	7.2	4.6	0.50	0.29^*^	0.20^*^	0.24^*^	0.31^*^
Thiamin (mg/d)	0.96	0.65	0.66	0.25^*^	0.36^*^	0.33^*^	0.35^*^
Riboflavin (mg/d)	0.68	0.42	0.51	0.33^*^	0.44^*^	0.44^*^	0.49^*^
Niacin (mg/d)	7.6	3.4	0.48	0.23^*^	0.22^*^	0.20^*^	0.22^*^
Vitamin B_12_ (mg/d)	0.82	0.82	0.34	0.02	0.12	0.15^#^	0.14^*^
Folic acid (g/d)	44.8	27.4	0.11	0.30^*^	0.13	0.13	0.16^#^
Vitamin C (mg/d)	24.4	20.7	0.54	0.22^*^	0.24^*^	0.26^*^	0.37^*^
Vitamin A (μg/d)	170.8	129.3	0.28	0.28^*^	0.18^#^	0.29^*^	0.33^*^
Vitamin D (μg/d)	1.82	3.6	0.12	−0.04	0.11	0.08	0.09
**MAR**	**-**	**-**	**0.39**	0.45^*^	0.48^*^	0.50^*^	0.58^*^

^*^p < 0.01, ^#^p < 0.05.

**Figure 2:**
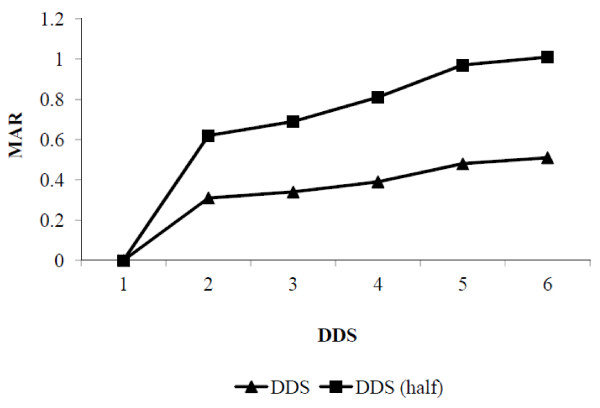
Distribution of mean adequacy ratio (MAR) with different dietary diversity scores (DDS).

We observed that mean MAR increased with increasing both DDS and DDS-half serving (Figure 2). This increasing trend explains nutrient adequacy was increasing directly with increasing food groups. Mean MAR increased more with DDS-half serving than DDS. Table 4 shows the extent to which FVS and DDS determine the MAR. MAR increased while increasing FVS and DDS. It shows that the FVS needs to be at least 9 in order to give a MAR above 0.45. Similarly, DDS needs to be at least 5 in order to give a satisfactory MAR.

**Table 4:** Mean MAR scores for different levels of FVS and DDS

**DDS**		**FVS**		
	**3-5**	**6-8**	**9-11**	**12-14**
3	0.27	0.34	0.34	0
4	0.25	**0.38**	**0.38**	0
5	0	**0.41**	**0.47**	**0.39**
6	0	0	**0.49**	**0.46**

#### Sensitivity and specificity analysis

**Figure 3:**
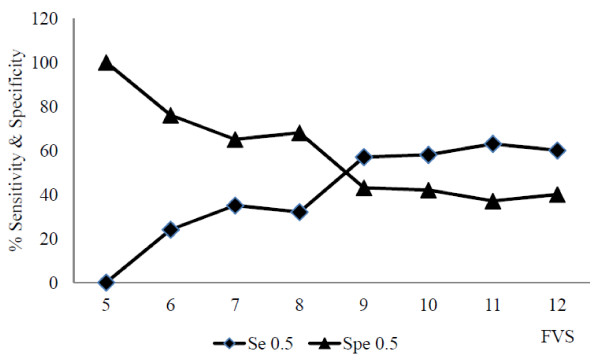
Sensitivity and specificity of FVS for 0.5 MAR for elders.

**Figure 4:**
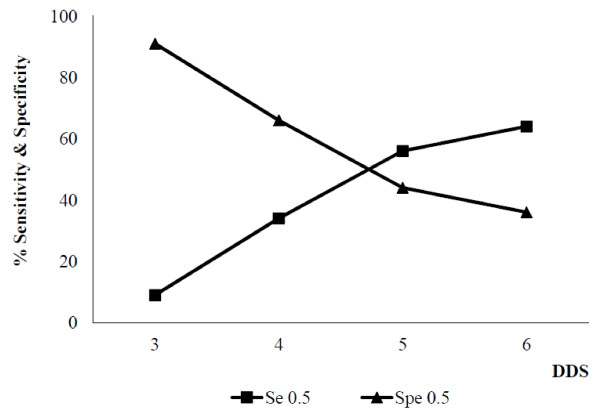
**Sensitivity and specificity of DDS for 0.5 MAR for elders.** [Se = Sensitivity, Spe = Specificity, Sensitivity indicates the % of elders truly at risk of nutrient inadequacy and identified as at risk. Specificity identifies the % of elders correctly identified as not at risk of inadequate intake].

Sensitivity indicates the percentage of elders who were really at risk (low MAR) who were correctly classified by low DDS. Specificity maximizes the percentage of elders not at risk of nutrient inadequacy (high MAR) and who are correctly classified by high DDS. In Figure 3, the best cut-off point for FVS was tested for sensitivity and specificity against the nutritional adequacy as MAR = 0.5 and the best cut-off point for maximizing both sensitivity and specificity was FVS of 9. In Figure 4, different cut-off points of DDS were tested as same as for FVS against MAR = 0.5. Using MAR of 0.5, the best cut-off point for maximizing both sensitivity & specificity was DDS of 4.5.

## Discussion

Lack of dietary diversity is particularly a considerable problem among poor populations of developing world as their diets are predominantly based on starchy staples [1]. In the present study, cereal consumption was 100% and almost all other food group consumption was low. Especially, their animal food consumption was poor. Furthermore, carbohydrate was the main energy contributor (68%) to the diet than other macronutrients. This condition may be due to their daily consumption of rice based diet with few other food commodities. Present study mainly focused on validation of dietary diversity indicators as an indicator of the nutrient adequacy of rural elderly people in Sri Lanka. As dietary diversity measures, simple count of individual food items (FVS), simple count of food groups (DDS) and dietary serving score (DSS) were used. In DDS, DDS and DDS-half serving scores were used separately. The average (standard deviation) of the food variety score (FVS), dietary diversity score (DDS), DDS-half and dietary serving score (DSS) was 8.4 (2), 4.4 (0.9), 3.8 (1.0) and 11.4 (2.5), respectively. Any of those scores were not up to their theoretical maximum. It reflects that their dietary diversity was not up to the optimum level. FVS counts all the food items consumed over the previous 24-h of survey date. When computing FVS, both beverages and condiments were not considered. Among the beverages we only excluded tea and coffee without milk i.e. milk and coffee infusion as the frequency of consumption of tea and coffee is high among the Sri Lankans and therefore to avoid overestimation of diversity of the diet. FVS could vary from the maximum value of 15 to minimum value of zero. Indeed, when computing FVS, we count the individual food items eaten without considering the group of the food. Therefore considering only FVS alone can therefore give a falsely favorable impression of the quality of diet. When constructing DDS, both nutritional aspects and local food group culture were considered. In the present study, six food groups for the DDS were adapted, since consumption of animal protein sources was low, animal (meat/fish/dairy) and plant protein sources (legumes/lentils) were taken as two different food groups. One of the limitations of this study is we excluded the counting of food group such as oils and fats and sugar and sweets. In DDS-half serving, other than just counting of food group, consumption of at least a half serving of that specific food was considered. When comparing to DDS, DDS-half serving improved the association with MAR, indicating that the performance of dietary diversity as an indicator of adequate nutrient intake is improved when a minimum intake for each food group was considered. This finding has important implications for field use of the indicator, it highlighted that the importance of considering the quantity consumed. In most field survey, we used to record simply the number of food groups consumed than information on quantities of food consumed. Moreover, it is important to identify which food group of their daily consumption may be the major contributor to the nutrition adequacy. Table 5 shows the mean dietary serving scores of each food group. The lower mean of the diversity score was related to fruit group and the higher one was for the cereals/roots group. For cereals/roots group, DDS was 4 and that was same as the theoretical score of 4 and other all food group scores were below the recommended serving score. It indicates that they could not achieve daily recommended serving sizes of these food groups considered in the present study to fulfill the nutrient adequacy. Sri Lankan Food Based Dietary Guidelines were used to adapt serving scoring system in this study [13]. According to the serving scoring system, the mean DSS was about 11 out of 20. It showed the poor diversity of the diet among this elderly population.

**Table 5:** The mean dietary serving score (DSS) within the food groups in the study population

**Food group**	**Mean**	**SD**
Cereals/roots	4	0.2
Vegetables	2.6	1.3
Fruits	1.0	1.4
Legumes/lentils	1.1	0.8
Meat/fish/egg	1.4	0.8
Milk/dairy products	1.3	1.2
**Total DSS**	**11.4**	**2.3**

Validation studies were performed according to the several criteria because validated dietary diversity indicators can be used as a precious and reliable nutrition tool in relation to health promotion and evaluation. As gold standards of nutrient adequacy, both NAR and MAR were used. For NAR values of nutrients, mean values were taken because intake of most of the nutrients was normally distributed. In the present study, mean MAR was about 0.4. The ideal cut-off point should be 1. It is convinced that the all nutrient requirements have been covered. But according to the results, only about 40% of the requirement of total nutrients has been covered. That may be the result of the diets that they were taken, because those diets were poor both in quality and quantity. In the present study, all four dietary diversity measures were significantly correlated with MAR, illustrating the potential of simple scores of dietary diversity for use as indicators of nutrient adequacy of the diet. These findings are similar to those of previous studies, testing the utility of dietary diversity as an indicator of nutrient adequacy in the diet of children, adolescents and adult women [1-3,15]_._

In furthermore validation, dietary diversity indicators were validated against nutrient NARs. Vitamin B_12_, vitamin D and folate were not significantly correlated with FVS, DDS and DDS-half serving. Vitamin B_12_ is found only in animal source foods, particularly liver, dairy products and eggs. The best sources of vitamin D are dairy products, legumes and green leafy vegetables and folate can be found in both animal source foods and plant based foods. Meat, egg, green leaves and dairy were the least consumed food groups in the study sample and lack of these groups could explain the poor correlation with DDS. Although they consumed legumes and fish commonly, the portion size consumed tended to be small.

An additional methodological contribution to validation of study was the sensitivity-specificity analysis and that was carried out to identify best cut-off points for predicting nutrient adequacy for both diversity indicators. Similar to the study done by Kennedy et al. 2007 [15], results of this study found the best cut-off points to maximize sensitivity and specificity was FVS of 9 and DDS of 4.5. Determining a fixed cut-off point, where elders can be defined as having greater or less risk of inadequate nutrient intake has potential application in immediate population nutrition assessment.

## Conclusion

The findings of this study confirm that the both the simple count of food items and food groups can be used as proxy indicators of nutrient adequacy of rural elderly population in Sri Lanka. Indeed, we found that the performance of the indicators is improved when considering the quantities of food consumed. However, the decision about the level of detail to incorporate into a programme or survey will depend on the resources availability and purpose of which the indicator is used.

## Abbreviations

DD: Dietary Diversity; FVS: Food Variety Score; DDS: Dietary Diversity Score; DSS: Dietary Serving Score; NAR: Nutrient Adequacy Ratio; MAR: Mean Adequacy Ratio; DS: Divisional Secretariat.

## Competing interests

The authors declare that they have no competing of interests.

## Authors’ contributions

KMR participated in the design of the study, data interpretation and draft the manuscript. PAEM contributed to the data collection, data analysis and coordination of the study. KDRRS assisted in data interpretation and critically revision of the manuscript. All authors read and approved the final manuscript.
